# Q-TWiST analysis of lapatinib combined with capecitabine for the treatment of metastatic breast cancer

**DOI:** 10.1038/sj.bjc.6604501

**Published:** 2008-08-19

**Authors:** B Sherrill, M M Amonkar, S Stein, M Walker, C Geyer, D Cameron

**Affiliations:** 1 RTI Health Solutions, Research Triangle Park, NC, USA; 2 GlaxoSmithKline, Collegeville, PA, USA; 3 GlaxoSmithKline, Greenford, UK; 4 Allegheny Cancer Center, Pittsburgh, PA, USA; 5 University of Leeds, Leeds, UK

**Keywords:** lapatinib, breast cancer, Q-TWiST, quality-adjusted survival

## Abstract

The addition of lapatinib (Tykerb/Tyverb) to capecitabine (Xeloda) delays disease progression more effectively than capecitabine monotherapy in women with previously treated HER2+ metastatic breast cancer (MBC). The quality-adjusted time without symptoms of disease or toxicity of treatment (Q-TWiST) method was used to compare treatments. The area under survival curves was partitioned into health states: toxicity (TOX), time without symptoms of disease progression or toxicity (TWiST), and relapse period until death or end of follow-up (REL). Average times spent in each state, weighted by utility, were derived and comparisons of Q-TWiST between groups performed with varying combinations of the utility weights. Utility weights of 0.5 for both TOX and REL, that is, counting 2 days of TOX or REL as 1 day of TWiST, resulted in a 7-week difference in quality-adjusted survival favouring combination therapy (*P*=0.0013). The Q-TWiST difference is clinically meaningful and was statistically significant across an entire matrix of possible utility weights. Results were robust in sensitivity analyses. An analysis with utilities based on EQ-5D scores was consistent with the above findings. Combination therapy of lapatinib with capecitabine resulted in greater quality-adjusted survival than capecitabine monotherapy in trastuzumab-refractory MBC patients.

Lapatinib (Tykerb/Tyverb) combined with capecitabine (Xeloda) has been shown to significantly delay disease progression, as compared with capecitabine alone, in women with HER2+ (ErbB2+) metastatic breast cancer that has progressed after standard therapies of anthracyclines, taxanes and trastuzumab (Geyer *et al*, 2006; Cameron *et al*, 2008). The combination therapy of lapatinib with capecitabine resulted in similar types of adverse events (AEs), and no increase in serious toxic effects, as compared with capecitabine monotherapy. These previously reported analyses focus on efficacy and safety outcomes independently. In a cancer setting it becomes important to compare treatment risks and benefits within a single metric.

We therefore conducted a quality-adjusted time without symptoms of disease or toxicity of treatment (Q-TWiST) analysis, to better estimate the overall benefit for patients using a single metric that incorporates progression, survival, treatment toxicities and quality-of-life. Originally developed for evaluating breast cancer treatments, this quality-adjusted survival method describes both the quality and quantity of survival time using a single metric (Gelber and Goldhirsch, 1986; Gelber *et al*, 1991). Q-TWiST compares the relative therapeutic value of treatments based on the patient experience within the context of clinical outcomes related to cancer and its treatment. Thus, Q-TWiST provides a measure of clinical benefit not necessarily apparent from separate efficacy and safety assessments. Assessments such as time to disease progression measure the length of time between clinical events but do not account for the value of that time to the patient.

The Q-TWiST method assumes that cancer patients progress through a set of health states of varying utility value for the individual patient. The health states most commonly used for Q-TWiST analyses of cancer treatment studies include: toxicity (TOX), time spent with toxic effects of study treatment; TWiST, time period without either symptoms of disease progression or toxicity; and relapse (REL), time following disease progression until death or end of follow-up (Cole *et al*, 2001).

Quality-adjusted survival is an understandable concept and can help individual patients make informed treatment decisions based on the relative importance they place on different health outcomes. Comparison of the amount of time a patient can expect before relapse without debilitating toxicities is a valuable aid in choosing between treatments. In this particular application, we were interested in showing whether patients on the combination of lapatinib and capecitabine would experience on average more time in a better health state compared with patients on capecitabine alone.

## Materials and methods

### Data source

The data source for this analysis was the previously reported Phase III clinical trial of lapatinib combined with capecitabine *vs* capecitabine alone in women with advanced or metastatic HER2+ breast cancer who had progressive disease following prior therapy which included an anthracycline, a taxane and trastuzumab (Geyer *et al*, 2006; Cameron *et al*, 2008). Subjects were randomised to receive either of the following treatments:

- Lapatinib 1250 mg daily without interruption and capecitabine 2000 mg m^−2^ per day, days 1 through 14, every 21 days; or
- Capecitabine 2500 mg m^−2^ per day, days 1–14, every 21 days.

Study enrollment was stopped early based on the unanimous recommendation of the independent data monitoring committee following a planned interim analysis. Based on independently reviewed imaging, the primary end point of time-to-progression had exceeded the predetermined stopping criteria, and review of toxicity data indicated no substantive safety concerns. The study closed to recruitment on 3 April 2006, when the results of the interim analysis were released and patients on monotherapy were offered the option of receiving the combination therapy. The analysis presented here uses data as of the lock date on 3 April 2006; thus, it does not take into account benefits that may have accrued beyond the end of the study.

### Definitions

#### Toxicity

In the primary analysis, the toxicity (TOX) state included all days spent with Grade 3/4 AEs after randomisation and prior to disease progression. A day with multiple AEs was only counted once. The TOX state comprised the total number of days spent with AEs, regardless of when AEs started or whether gaps occurred between AEs. Per convention, AEs occurring after progression were not included in the TOX state. Expanded definitions of the TOX state were examined in sensitivity analyses.

#### Progression-free survival

The end of the time period without symptoms of toxicity or disease progression (TWiST) is based on progression-free survival (PFS), including events of disease progression and deaths due to any cause. For subjects who did not progress or die as of the last data date, PFS was censored at the time of the last independently assessed radiological scan preceding the initiation of any alternative anticancer therapy. If a subject had only a baseline visit or did not have an independently reviewed radiological scan dated prior to initiation of alternative anticancer therapy, PFS was censored at the date of randomisation.

#### Utility weights

For the threshold utility analysis, a matrix of hypothetical utility weights (*u*_*i*_) for the TOX and REL health states was constructed by varying utility from 0 to 1 by 0.25, resulting in 25 combinations, relative to the utility of TWiST. For treatment comparison purposes, TWiST is used as the reference state with utility set equal to 1, representing the highest utility that can be expected for a patient with metastatic breast cancer.

Additionally, individual utilities were determined from patient-reported health status on the EQ-5D (EuroQol Group, 1990), a simple and validated questionnaire measuring five dimensions: mobility, self-care, usual activities, pain/discomfort, and anxiety/depression. The EQ-5D was scheduled to be completed predose on day 1, every 6 weeks for the first 24 weeks, followed by every 12 weeks thereafter, and at withdrawal from the randomised therapy. Patient-reported utility weights were derived from the EQ-5D using published algorithms (AHRQ, 2005). The maximum possible value on the EQ5D is one; utility values less than zero represent a state evaluated as worse than death for the patient.

### Statistical analysis

#### Estimation of health-state durations

The Product-Limit Method (Kaplan and Meier, 1958) was used to estimate the mean amount of time in the following states:

- With toxicities after randomisation but prior to progression (i.e., TOX),
- From randomisation to progression or death (i.e., PFS), and
- From randomisation until death from any cause (i.e., overall survival (OS)).

Survival curves corresponding to toxicity duration, PFS, and OS were plotted on a single graph for each treatment group. The areas between the curves represent the restricted mean durations of TWiST and REL as follows: 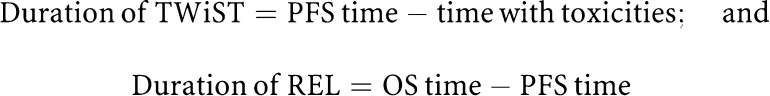


All patients in this study had previously relapsed, so the REL state here refers to the period after further progression. To look over the same timeframe for both treatments, analyses were restricted to the median overall follow-up time. This convention is required as analyses represent areas under the curve; both treatments are evaluated over the same period of time. The primary analysis was performed on the intent-to-treat (ITT) population, with TOX defined to include Grade 3/4 AEs. The results of this analysis are reported unadjusted and then are employed during the Q-TWiST calculation.

#### Calculation of Q-TWiST and threshold utility analysis

Q-TWiST for each treatment arm was calculated as follows: 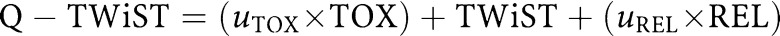 where TOX, TWiST, and REL represent the mean estimated health-state durations, and *u*_TOX_ and *u*_REL_ denote utility weights for the TOX and REL states, respectively. Note that Q-TWiST equals the mean OS when *u*_TOX_=*u*_REL_=1 and equals the mean PFS when *u*_TOX_=1 and *u*_REL_=0.

A threshold utility analysis was performed to illustrate which utility combinations are expected to result in different durations of quality-adjusted survival between treatment groups. Differences in mean Q-TWiST between treatment groups were calculated for each combination of hypothetical utility weights (Cole *et al*, 2001). Ninety-five percent confidence intervals for the mean differences and two-sided *P*-values for testing the null hypothesis of no difference were performed, based on the normal approximation, with s.e. calculated by bootstrapping (Glasziou *et al*, 1990). Points between selected utility values can be interpolated from the threshold utility graph.

#### Sensitivity analyses

In two sensitivity analyses, the TOX state was redefined to include (1) all AEs of any grade or (2) AEs of any grade classified as treatment-related according to the protocol.

#### Incorporation of observed utility data

For each patient, the average utility weight derived from EQ-5D assessments during a health state was assigned as a per-person utility weight for the TOX, TWiST, and REL states. If a utility weight for the TWiST state was not available for a patient, then the predose EQ-5D score was used. For patients who progressed (i.e., were not censored for progression), the last EQ-5D score after progression was used for the REL state. Patients who died on the date of progression were assigned utility=0 for the REL state. To compare groups, the overall average utility relative to the average reported utility for the TWiST state was used.

## Results

As of 3 April 2006, 399 patients were enrolled and randomised either to lapatinib plus capecitabine (*n*=198) or to capecitabine alone (*n*=201). The ITT population includes all randomised patients, who were well balanced across treatment groups. All patients had advanced or metastatic HER2+ breast cancer and had progressive disease following prior therapy, which included an anthracycline (97%), a taxane (97%) and/or trastuzumab (97%) Almost all patients had metastatic disease (96%), with 78% involving visceral lesions and 49% having lesions at three or more sites.

During the entire study period, most patients experienced at least one AE (96% in the lapatinib plus capecitabine group and 89% in the capecitabine only group). In the combination group, 38% (76 of 198) of patients experienced a Grade 3/4 AE prior to progression or censoring for progression, with an average start time of 67 days after randomisation. In the capecitabine only group, the percentage was 36% (73 of 201), starting on average within 55 days of randomisation.

**Figure 1:**
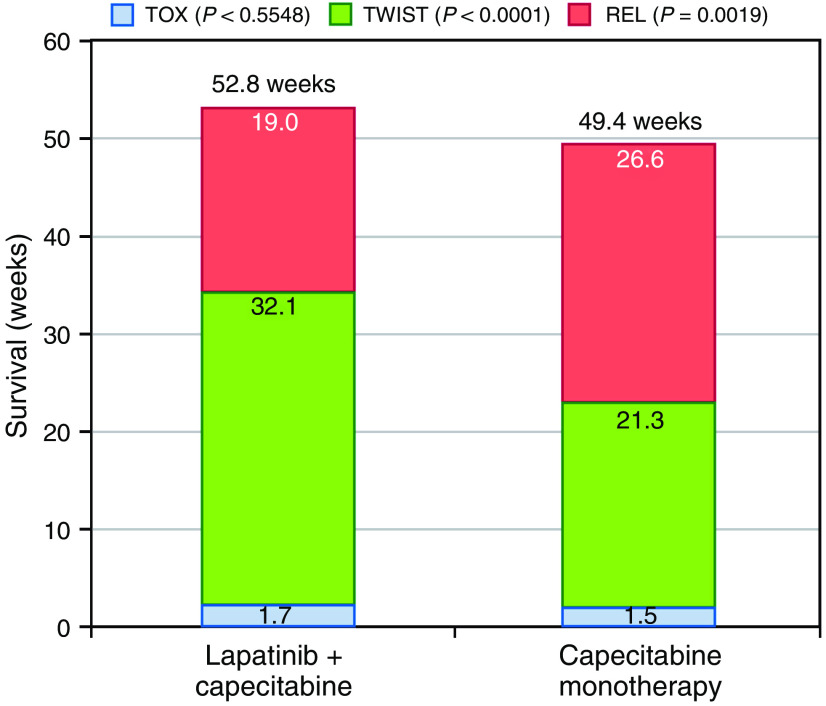
Unweighted mean duration of health states (weeks). TOX=toxicity state includes days with severe and life-threatening adverse events prior to progression only; TWiST=time without symptoms or toxicity; REL=relapse period until death or end of follow-up.

**Figure 2:**
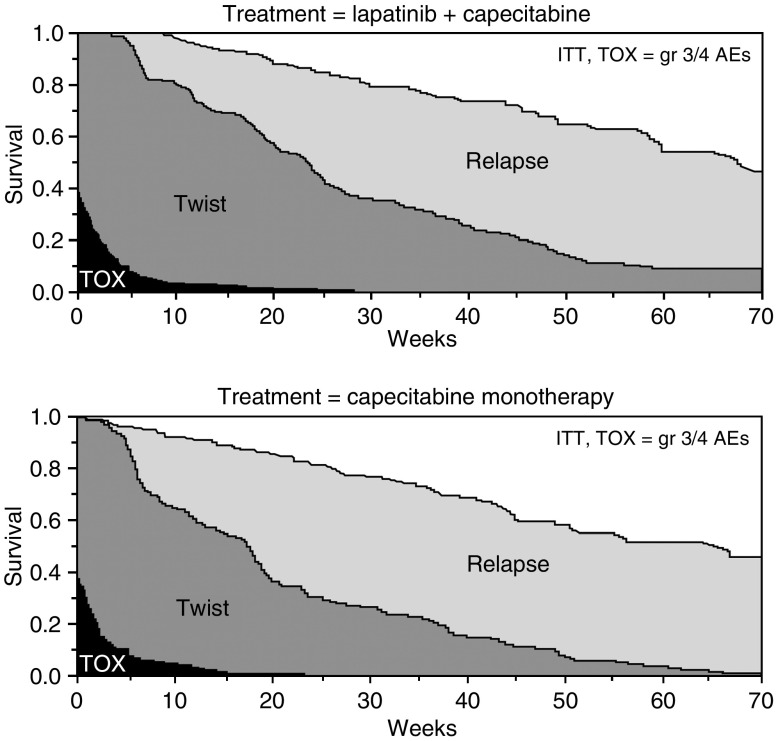
Partitioned survival curves. AEs=adverse events; ITT=intent-to-treat; TOX=toxicity; TWiST=time without symptoms or toxicity.

Figure 1 shows the unweighted mean duration of each health state. Overall median follow-up for survival was approximately 67 weeks. Average duration with Grade 3/4 AEs (prior to progression) was less than 2 weeks, and the difference between groups was not statistically significant. Mean time in the TWiST state (time without severe toxicities or symptoms of progression) comprised the primary difference in survival time between the treatment groups. The additional 3.5 weeks of overall survival for the combination group *vs* the monotherapy group was more than accounted for by the difference in TWiST (32.1 weeks *vs* 21.3 weeks, *P*<0.0001). Partitioned survival plots appear in Figure 2.

**Figure 3:**
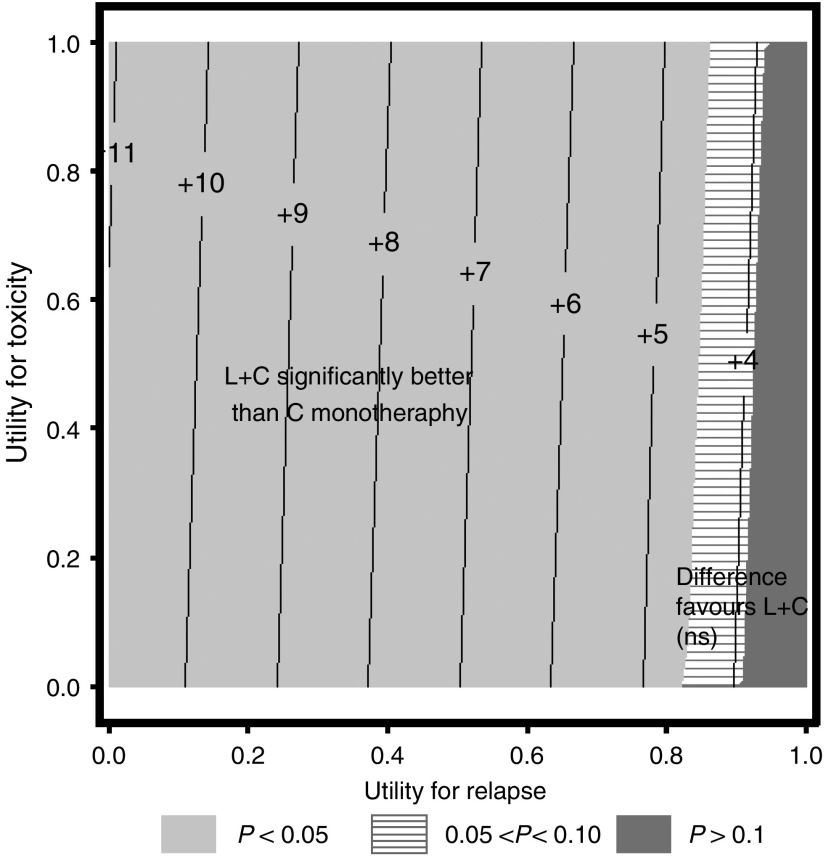
Threshold utility plot (Q-TWiST differences in weeks). Positive numbers indicate longer Q-TWiST for patients on combination therapy. Shading represents combinations of utility values for which the difference in Q-TWiST is significant: medium grey, *P*<0.05; light band, 0.05<*P*<0.10; dark grey, *P*>0.10. AEs=adverse events; ITT=intent-to-treat; Q-TWiST=quality-adjusted time without symptoms of disease or toxicity of treatment; TOX=toxicity; ns=not significant.

In the primary analysis, the difference in quality-adjusted survival time between combination and monotherapy ranged from 3 to 11 weeks (Table 1), depending on relative valuations of the health states. Differences were statistically significant except where REL was assumed to have a utility equal to TWiST. Thus, any patient who places less than full utility on time after progression is expected to gain more quality-adjusted survival with lapatinib, regardless of what value the patient places on the TOX state. When all states are valued at full utility (last row of Table 1), the mean survival advantage is 3.5 weeks (*P*=0.1556) although the survival data is not yet fully mature. The threshold plot in Figure 3 illustrates the Q-TWiST differences between groups, with varying combinations of utility weights for TOX and REL.

As EQ-5D can produce negative utility values, we also ran the analyses using negative utilities for the TOX and REL states. All comparisons resulted in a positive Q-TWiST advantage for the L+C combination *vs* C monotherapy. Negative utilities on the TOX state diminished the advantage, whereas negative utilities on the REL state increased the advantage. In other words, the less value a patient places on time after progression, the more the combination therapy is expected to increase quality-adjusted time.

In the sensitivity analyses with TOX expanded to include all AEs or treatment-related AEs of all grades, differences were slightly less pronounced; patients in the combination group experienced AEs for 4–5 weeks longer than patients in the single-treatment group. However, the quality-adjusted survival advantage of lapatinib plus capecitabine remained statistically significant, with most utility–weight combinations resulting in a Q-TWiST advantage for the lapatinib plus capecitabine combination. This finding remained despite the fact that lower grade AEs were not differentiated from more serious AEs and utility values were applied uniformly to the entire TOX period.

Average observed utility values for each health state are shown in Table 2. Although 94% of patients completed at least one EQ-5D assessment, averages are based on a smaller subset of patients. Visits at which EQ-5D assessments were taken often did not correspond to the timing and conditions of a given health state. Consequently, observed utility scores for the TOX state were available for only 30% of patients who experienced a Grade 3/4 AE. Utilities for the REL state were based on about 30% of patients in the study.

The overall average utility value observed during the TOX state (Grade 3/4) was 0.59, and this value was similar between groups. Patient-reported utility weights for the TWiST states were less than one on average, consistent with the poor health of these patients even prior to progression. The TOX utility values ranged from −0.6 to 1.00, and REL utility values ranged from −0.23 to 1.00. Overall observed utilities in TOX and REL were normalised relative to a utility=1 for TWiST, providing an average relative utility of 0.90 for TOX and 0.65 for REL. On the basis of these values, the between-group Q-TWiST difference was 6.1 weeks (*P*=0.006, Table 1). Findings were similar when this analysis was repeated to include only patients who contributed at least one EQ-5D value.

## Discussion

In the primary analysis, all hypothetical utility combinations resulted in greater quality-adjusted survival times for the women randomized to lapatinib plus capecitabine compared to capecitabine only. The difference was statistically significant, except when the utility assigned to the REL state was more than 75% of the utility of the TWiST state, a value greater than the average for the patients' own assessments in this study. Given that patients have their own ways of valuing their time, the grid of possible values allows an individual physician or patient to estimate what their own experience will be using their particular circumstances and set of values. For patients who rate the utility of both TOX and REL at half the utility of TWiST, the combination treatment is expected to provide an additional 7 weeks of quality-adjusted survival time compared with capecitabine alone over a 67-week time horizon. These results showing a quality-adjusted survival advantage for lapatinib with capecitabine compared with capecitabine alone were robust for the inclusion of varying grades of toxicity. As less serious events, which were more common on the combination arm, were given the same utility rating as more serious ones, this sensitivity analysis is conservative and may underestimate the magnitude of the quality-adjusted survival advantage presented by combination therapy.

Revicki *et al* (2006) recommend that a clinically important difference for Q-TWiST is approximately 10% of overall survival in a study. Differences as low as 5% may be important for some diseases, and differences as great as 15% are clearly important. In this study, Q-TWiST is between 3 and 11 weeks longer (4.5–16% of median OS) for subjects on combination therapy than for subjects on monotherapy, depending on relative utility weightings of the health states. When utilities for periods of toxicity and relapse were valued at half that of TWiST or when using observed patient utility data, the Q-TWIST difference was approximately 7 weeks, approximately 10% of the median overall survival of 67 weeks, which would be considered clinically important according to the Revicki guideline.

In sensitivity analyses, with the TOX state definition expanded to include treatment-related AEs or all AEs, the TOX state was longer for the combination group, as would be expected. However, the quality-adjusted survival advantage of lapatinib plus capecitabine remained evident for almost all hypothetical utility combinations. Most differences were statistically significant between groups. Differences were less pronounced only if REL was valued at the level of TWiST or if REL was valued almost at the level of TWiST and higher than TOX.

Patient-reported utility data from the trial was used to estimate average utility weights for the health states. However, there are limitations on basing quality-adjusted survival on actual patient experience of the treatments administered. EQ-5D data were not reported on all patients or at all visits, and fewer than half of the patients had an end-of-study assessment. Furthermore, to use EQ-5D data to ascribe utilities to the TOX state, the assessment should have taken place on a day when the AE was being experienced, and this was uncommon. REL utilities were based on values collected at withdrawal from study treatment and could be determined only for patients who experienced the progression event, not for those who were censored for the event.

Despite these limitations, the overall average reported utility for REL was 0.43, which is in the 0.41–0.69 range reported by Earle *et al* (2000) for progressive metastatic breast cancer. At this level, and with average utility for TOX and TWiST at 0.59 and 0.66, respectively, the lapatinib plus capecitabine combination treatment used in this study provides, on average, significantly more quality-adjusted survival time than does treatment with capecitabine alone.

Earle *et al* (2000) also report utility in the range of 0.16–0.54 for terminal metastatic breast cancer, and Nooij *et al* (2003) cite a physician-based utility=0.23 for the progressive state in metastatic breast cancer. These lower values may reflect a more realistic range of values than the observed values for the entire period after progression, as the deterioration in health that eventually occurs may not be captured well by assessments that were taken close to the progression date. Application of lower utilities for the REL state would result in a more pronounced Q-TWiST advantage for the lapatinib plus capecitabine treatment combination.

In summary, the lapatinib plus capecitabine combination provided significantly greater Q-TWiST than did capecitabine alone. The full impact of the combination cannot be determined, because of the early closure to accrual and subsequent cross over, but it is likely that the average 7 weeks improvement is an underestimate of the overall benefit.

## Figures and Tables

**Table 1:**
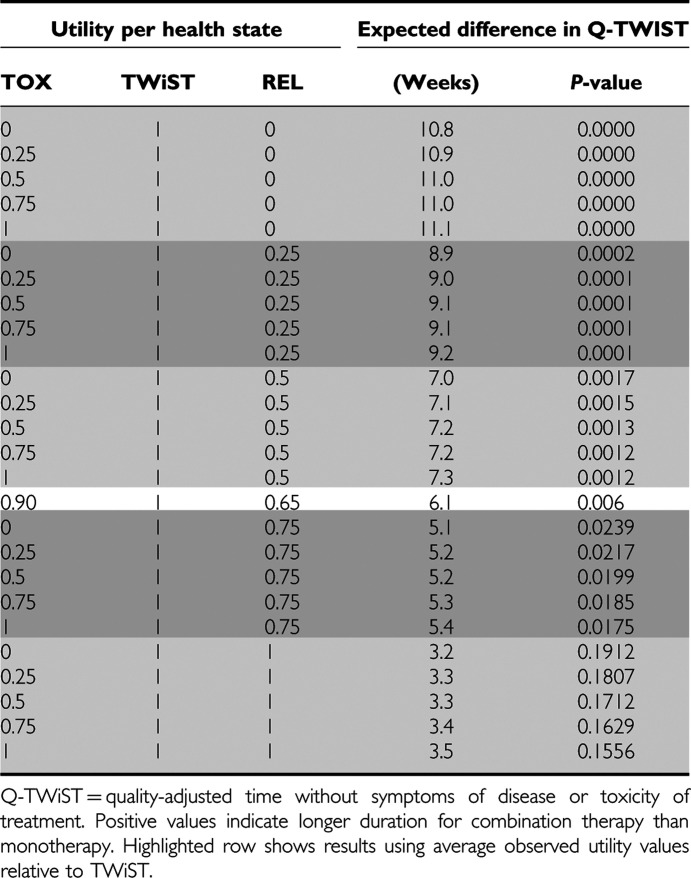
Q-TWiST differences for varying combinations of utility weights

**Table 2:** Average utility values by health state, based on EQ-5D scores

	**Lapatinib plus capecitabine (*N*=198)**		**Capecitabine monotherapy (*N*=201)**	
**Health-state ITT population**	***n***	**Utility**	***n***	**Utility**
TOX: Grade 3/4	27	0.60	17	0.59
TWiST	168	0.66	157	0.66
Relapse	50	0.41	67	0.44
